# Role of circulating tumor DNA and cell-free DNA biomarkers in diagnosis and prognosis of oral cancer - a systematic review

**DOI:** 10.1186/s12903-025-05898-3

**Published:** 2025-04-11

**Authors:** Samrina Mohammad, Ihsan Ullah, Asif Ali, Zainab Jan, Benish Aleem, Muslim Khan, Waqas Naseem

**Affiliations:** 1 Department of Oral Pathology, Khyber College of Dentistry, Peshawar, Pakistan; 2 IPDM, Khyber Medical University, Peshawar, Pakistan; 3 Department of Pathology, College of Medicine, Qassim University, Buraydah, Saudi Arabia; 4 Institute of Radiation and Nuclear Medicine IRNUM Hospital, Peshawar, Pakistan; 5 Maxillofacial Surgery, Khyber College of Dentistry, Peshawar, Pakistan; 6 Department of Health Sciences, University of Debrecen, Debrecen, Hungary

**Keywords:** Oral squamous cell carcinoma, Head and neck cancer, Prognosis, Diagnosis, CtDNA, CfDNA

## Abstract

**Background:**

Oral squamous cell carcinoma is the most common malignant neoplasm of the oral cavity, contributing significantly to cancer-related mortality worldwide. Circulating tumor DNA could be a promising biomarker for the early diagnosis and prognosis of oral cancer.

**Objective:**

The aim of this systematic review was to consolidate the existing literature on the role of circulating tumor DNA (ctDNA) and cell-free DNA (cfDNA) in the diagnosis and prognosis of oral cancer.

**Methodology:**

The review protocol followed PRISMA guidelines. A systematic search was conducted across PubMed, Web of Science, Google Scholar and SCOPUS. Only English-language studies were included, while narrative reviews, HPV-positive OSCC, systematic reviews, meta-analyses, abstracts, and letters to the editor were excluded. Data were extracted on study design, country, sample size, participant characteristics, assessment methods, type of oral cancer and measured outcomes. Risk of bias was evaluated using Newcastle-Ottawa Scale (NOS).

**Results:**

A total of 3,155 records were identified, out of which 17 studies met the inclusion criteria. These comprised eleven cohort studies, one was a case series, two were descriptive studies, and three were case-control studies. The studies primarily addressed oral squamous cell carcinoma (OSCC) and head and neck squamous cell carcinoma (HNSCC). Findings revealed that elevated cfDNA levels are associated with poor prognosis, lymph node metastasis, larger tumor size and advanced disease stages. ctDNA acts as a predictive tool for monitoring cancer progression, treatment response, recurrence risk, and overall survival. Among 12 studies evaluated using NOS, 8 were of good quality, while 4 were fair quality.

**Conclusion:**

ctDNA and cfDNA exhibit promising prognostic and diagnostic potential for OSCC and HNSCC. Elevated cfDNA levels correlate with poor prognosis, while ctDNA shows potential for monitoring cancer progression and treatment response.

## Introduction

### Background

Oral squamous cell carcinoma (OSCC), a primary oral cancer, frequently metastasizes and has a poor prognosis with high mortality rates due to its aggressive nature and local invasion [1]. Early detection remains challenging, often leading to late-stage diagnoses that limit treatment effectiveness. Improving OSCC outcomes requires addressing early detection challenges and developing more effective treatment strategies. Composite resection is common for patients with lymph node involvement [2]. Detecting OSSC early is difficult due to the unreliability of specific symptoms and diagnostic studies [3]. Widespread screening is not feasible and may only incidentally identify small tumors [4]. In clinical practice, post-surgical pathological staging predicts outcomes better than the current markers [5]. 

Circulating tumor DNA refers to DNA fragments shed by tumor cells into the bloodstream, whereas cell-free DNA consists of tumor-derived DNA circulating freely in bodily fluids. The presence of free circulating DNA (cfDNA) in the bloodstream is common among cancer patients, resulting from apoptotic and necrotic processes typical of tumor cells [6]. This phenomenon also occurs in individuals with benign diseases and healthy individuals. Researchers have explored both the quantity and quality of cfDNA as potential biomarkers for various tumors [7]. These biomarkers may prove useful for early detection, detecting minimal residual disease, monitoring treatment response, predicting prognosis and guiding treatment decisions in different types of cancers [8]. 

At the molecular level, cell-free DNA is released into the bloodstream through apoptosis, necrosis, or active secretion from both normal and tumor cells, while circulating tumor DNA (ctDNA) represents the tumor-derived fraction of cell-free DNA. ctDNA carries tumor-specific mutations, copy number variations, and DNA methylation patterns, reflecting the tumor’s genomic profile [9]. In oral squamous cell carcinoma, common alterations include TP53 and PIK3CA mutations, along with hypermethylation of p16 and RASSF1A genes. Quantitative levels of cell-free DNA may indicate tumor burden, while ctDNA analysis aids in early detection, treatment response monitoring, and prognosis prediction. However, due to its low concentration, particularly in early stages, highly sensitive techniques like polymerase chain reaction-based assays and next-generation sequencing are essential for reliable detection [10]. 

For oral cancer patients, studies have investigated ctDNA and cfDNA for detecting disease-associated mutations, assessing tumor burden, monitoring disease progression, and predicting response treatment response. However, despite significant potential, the clinical application of ctDNA and cfDNA in oral cancer remains under development, requiring further research to fully establish their diagnostic and prognostic roles and standardize methodologies for their use.

### Rationale

Circulating tumor DNA (ctDNA) and cell-free DNA (cfDNA) are emerging as potential biomarkers obtainable through blood samples, offering a less invasive alternative to traditional diagnostic methods. This review systematically examines existing literature to comprehensively understand the current state of research regarding ctDNA and cfDNA utilization in oral cancer. Investigating their diagnostic role aims to evaluate their efficacy in early detection, which is crucial for improving treatment outcomes. Additionally, assessing these biomarkers prognostic value seeks to determine their potential for predicting disease progression and patient outcomes. By conducting a systematic review, this study synthesizes available evidence, evaluates study quality, and identify trends or research gaps. This review may contribute to understanding the clinical utility of ctDNA and cfDNA in oral cancer, potentially influencing diagnostic and prognostic clinical practices.

### Objective

The objective of this systematic review was to summarize the existing literature on the role of ctDNA and cfDNA in the diagnosis and prognosis of oral cancer.

## Methodology

### Protocol

The review protocol followed the Preferred Reporting Items for Systematic Reviews and Meta-Analyses guidelines (PRISMA) [11, 12]. Any subsequent modifications were duly recorded. All data extracted were taken from already approved studies, so ethical approval was not required. This systematic review adhered to the Cochrane Handbook [12] and the PRISMA statement [12] for its conduct and reporting, respectively.

### Research question

The focused research question was: What is the diagnostic and prognostic role of circulating tumor DNA (ctDNA) and cell-free tumor DNA (cfDNA) in oral and head and neck cancer patients?

### Search strategy

An unrestricted search was conducted for retrieving relevant studies from four major databases: PubMed, Web of Science, Google Scholar and SCOPUS. In Google Scholar, the first 200 hits were searched. The search terms used were as follows:

((((prognosis) OR (diagnosis)) OR (survival)) AND ((((ctDNA) OR (cfDNA)) OR (circulating tumor DNA)) OR (cell-free DNA))) AND (((oral cancer) OR (oral squamous cell carcinoma)) OR (oral malignancy)).

All titles and abstracts were carefully screened and reviewed.

### Inclusion and exclusion criteria

- The study was independently reviewed by two authors (S.M, U.H) Studies were included in the review if they met the following criteria: (a) Studies conducted in English language; (b) Participant size greater than 20; (c) Blood samples of ctDNA were measured; (d) The detection of ctDNA was measured on the basis of quantification by Qubit or Nanodrop and Polymerase Chain Reaction (PCR); (e) Reporting prognosis and/or diagnosis of oral cancer patients using ctDNA or cfDNA as tumor markers.
  Studies were excluded if they were narrative reviews, HPV-positive OSCC (to have more homogeneous population, as HPV-associated tumors exhibit distinct molecular profiles that could influence biomarker outcomes), studies with insufficient data, reviews, case reports, meta-analyses, and letters to the editor.

### Data extraction and quality assessment

Initial screening was conducted for all studies retrieved from all databases, and duplications were removed in an Excel spreadsheet. After the removal of duplicates, screening of titles and abstracts were performed to eliminate irrelevant papers. The full texts of the remaining studies were assessed against the inclusions and exclusion criteria. Finally, studies that met the inclusion criteria were included in the quantitative and qualitative synthesis. Two authors independently conducted the study selection process, and any discrepancies were resolved through discussion. Data were extracted from the included studies for several key aspects: study setting, sample size, country, participant characteristics including gender (M/F) and mean age, method of assessment, type of oral cancer, outcome measured, and main findings. The Newcastle-Ottawa Scale (NOS) tool [13] was used to evaluate the risk of bias in the observational studies included in the research. Two authors independently assessed the risk of bias in the individual trials, and any differences were resolved through discussions. The NOS comprised three categorical criteria with a maximum score of 9 points. Studies were rated based on following scoring system: scores of more than or equal to 7 points were deemed “Good” quality, scores between 2 to 6 points were considered “Fair”, and scores of more than or equal to 1 point were classified as poor quality.

## Results

### Study selection

In this study, a total of 3,155 records were initially identified through electronic databases (PubMed, SCOPUS and Web of Science), supplemented by 1 record sourced from Google Scholar. Following a thorough screening process, 102 duplicate records were methodically eliminated, narrowing down the dataset to 3,054 unique records. Subsequently, through a rigorous evaluation of titles and abstracts, 2,993 records were deemed ineligible for inclusion based on predefined criteria, leaving 61 full texts for further scrutiny against the eligibility criteria.

**Fig. 1:**
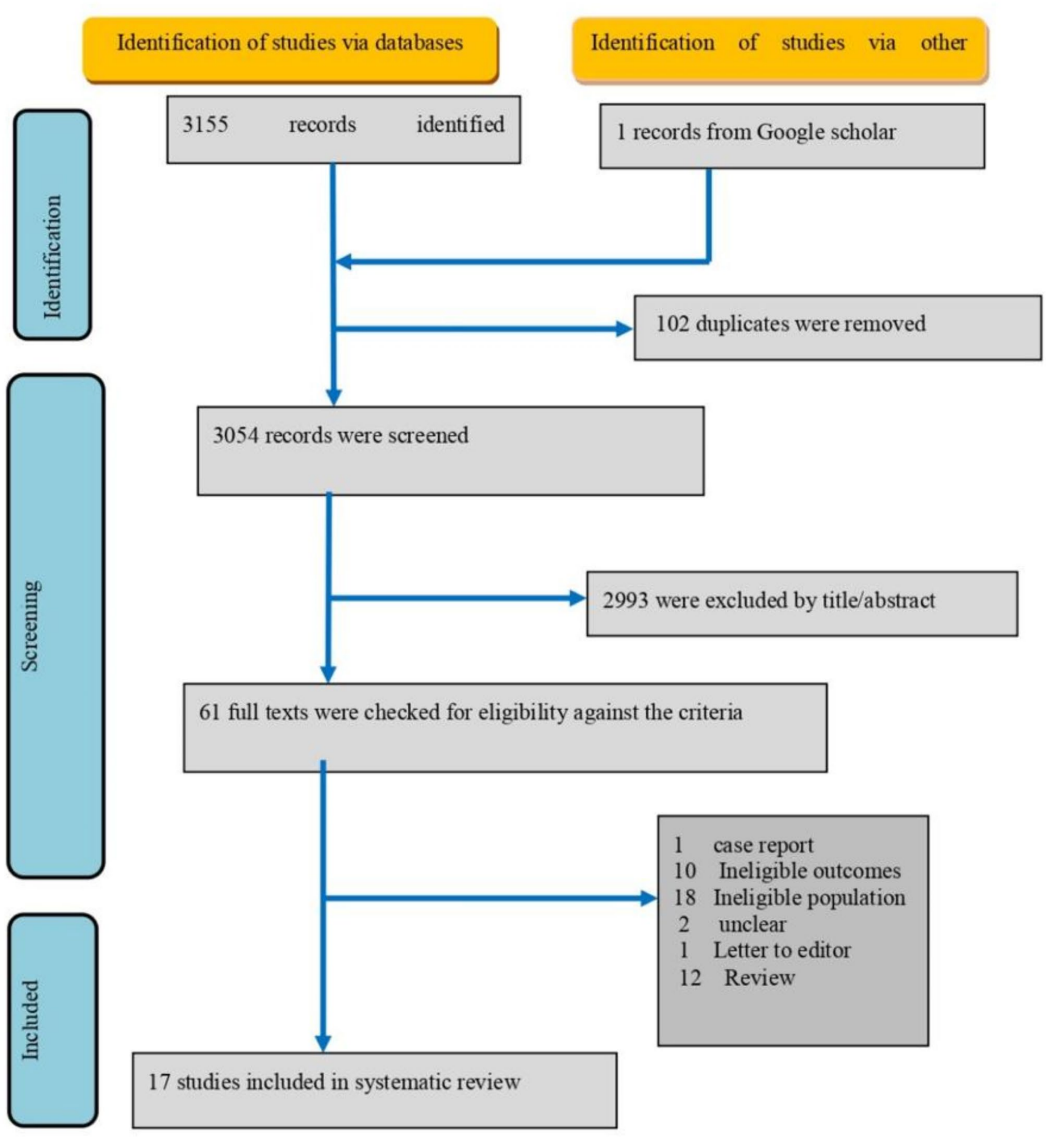
PRISMA flowchart 2020

Among these texts, our analysis revealed that 1 constituted a case report, while 10 were excluded due to ineligible outcomes. Additionally, 18 texts were disqualified as they pertained to an ineligible population, whereas 2 exhibited ambiguities regarding their classification. Furthermore, we identified 1 letter addressed to the editor and 12 records that were classified as reviews. The investigators finally agreed to include 17 studies deemed pertinent to our research objectives, as outlined by our rigorous inclusion criteria’s. (Fig. 1).

### Study characteristics

The characteristics of the included 17 studies are shown in the Table 1. Eleven studies were cohort, one was a case series, two were descriptive studies, and three were of case-control studies. These studies were conducted in various countries, including Taiwan, Japan, India, France, the UK, Belgium, Israel, the USA, Poland, Brazil, and Finland.

**Table 1:** Characteristics of the included studies

Study	Design/Setting; Country	Sample size	Characteristic mean Age; Gender(M/F)	Method of assessment	type of oral cancer	Outcome measured	Main findings
Lin et al. [6] 2018	case control/ Taiwan	121 OSCC patients, 50 controls	19.39 years; 44% /56%	Quantitative spectrometry	OSCC	cfDNA	Elevated cfDNA, correlated with tumor size, lymph node metastasis, and late stage; Poor prognosis associated with higher cfDNA levels
Hamana et al. [2] 2005	Cohort/ Japan	64 patients	68 years;38/26	Examination of DNA from normal and the tumorous tissue, serum DNA at the three time points (preoperatively, postoperatively, and 4 weeks postoperatively).	OSCC	ctDNA role in prognosis and recurrence	ctDNA could be a useful predictive tool for monitoring OSCC prognosis
Kakimoto et al. [16] 2008	Cohort/ Japan	20 patients	62.4 years; 11/9	Microsatellite blood assay using polymerase chain reaction (PCR)-based analysis	OSCC	serum DNA in Prognosis, recurrence, and distant metastasis	Microsatellite analysis offers a predictive tool in assessing risk of recurrence, metastasis and death.
Husain et al. [17] 2020	Cohort/India	25	NR/NR	Assessment of ctDNA levels in peripheral blood before and after surgery	OSCC	ctDNA role in aggressiveness of OSSC	Higher circulating tumor DNA (ctDNA) levels in patients with oral cancer are associated with age ≤ 40, female gender, short disease duration, infiltrative tumors, and lower tumor volume, and may complement biopsy/histopathological findings while indicating potential tumor aggressiveness.
Desai et al. [18] 2018	Cross sectional Observational/ India	44	NR/NR	Quantitative and qualitative assessment of cfDNA in plasma	Oral cancer	cfDNA in clinicopathologic parameters of OSCC	cfDNA correlated with nodal metastasis (*p* = 0.001) and clinical stages (*p* = 0.006);
Shukla et al. [19] 2013	Case-control study/ India	390 OSCC and 150 OED	OSCC: 53.16 years;78/22	Spectrophotometry and (NanoDrop ND-1000 spectrophotometer; Thermo Fisher Scientific) in sampled blood and plasma	OSCC	cfDNA level among OSCC and OED and its role in prognosis of these diseases	No significant differences in cfDNA levels in blood between groups. Disease progression in oral malignancy did not correlate with changes in cfDNA levels in blood or plasma.
Coulet et al. [20] 2000	Cohort study /France	117	NR;105/12	Microsatellite instability at UT5085 and p53 mutation screening	HNSCC	Presence of plasma tumor DNA	High level of ctDNA in Head and Neck Squamous Cell Carcinoma
Taylor et al. [21] 2023	Cohort study /UK	53	62 years^*^;40/13	Quantification of the plasma circulating tumor DNA (ctDNA) measured by Cancer Personalized Profiling	Recurrent/metastatic head and neck HNSCC	Role of ctDNA survival of patients	Baseline ctDNA abundance was not linked to overall survival (OS) or progression-free survival (PFS)
Chikuie et al. [22] 2022	Cohort/Japan	20	65 years^*^;16/4		HNSCC	Recurrence and survival	Detecting ctDNA during follow-up can help predict respond to treatment and recur.
Honoré et al. [23] 2023	Cohort /Belgium	53	63.7 years^*^;40/13	26-gene next-generation sequencing panel in plasma sample	HNSCC	Progression-free survival and overall survival	ctDNA effectively predicts disease progression and survival without the need for tumor sequencing.
Kampel et al. [24] 2023	Cohort study/ Israel	70 patients	65 years; 41/39	Next-generation sequencing (NGS) to detect TP53 mutation	Head and neck squamous cell carcinoma (HNSCC)	Progression-free survival	Detection of mutated TP53 ctDNA were associated with the poor progression-free survival and the regional metastases. Detectabley ctDNA correlated with regional spread and the poorer 5-year progression-free survival. High-risk worst pattern of the invasion (WPOI grade 4–5) and deep invasion was frequently found in the patients with detectable ctDNA.
Egyud et al. [25] 2018	Cohort study/ USA	8	NR/NR	Patient-specific tumor sequencing to identify mutations; testing for circulating tumor DNA in plasma	HNSCC	Recurrence and monitoring	ctDNA shows potential for monitoring disease recurrence and guiding treatment strategies in head and neck cancer.
Kumari et al. [26] 2022	Case Control study/ India	total 202 cases with malignancies (68 OSCC)	NR/NR	Quantification of cfDNA using real- PCR and beta-globin gene amplification	OSCC	cfDNA concentrations and its correlation with cancer stages (T, N, M)	cfDNAsignificantly increased with higher cancer stages in OSCC.
Mazurek et al. [27] 2016	Cohort Multi-center/Poland	200	59 years^*^;40/160	TaqMan-based TERT amplification	HNSCC	cfDNA levels	higher cfDNA in clinical N2–N3 disease and stage IV cancer;
Nunes et al. [28] 2001	Descriptive study/ São Paulo, Brazil	91	NR/NR	Polymorphic microsatellite markers assay	HNSCC	Detection of ctDNA in plasma	ctDNA has potential for early diagnosis of head and neck tumors.
Sandra Perdomo et al. [29] 2017	Case series/France	2	NR/NR	Targeted screening of ctDNA mutations	HNSCC	ctDNA mutations	ctDNA mutations has not associated with overall survival of HNSCC
Silvoniemi et al. [30] 2023	Cohort/ Finland	26	NR/NR	Liquid biopsy techniques	HNSCC	Detection of ctDNA	positive association between high metabolic tumor burden and the detection of ctDNA

OSCC, oral squamous cell carcinoma; ctDNA, circulating tumor DNA; cfDNA, cell free DNA; M/F, males/females; NR, not reported; OED, oral epithelial dysplasia; HNSCC, Head and neck squamous cell carcinoma;

^*^Median

The sample sizes in the studies varied, with the number of participants ranging from as few as 2 to as many as 390 oral squamous cell carcinoma (OSCC) patients. Some studies included head and neck cancer patients. Of the studies reviewed, nine reported information on the participants’ age and gender. The reported mean ages ranged from 19.39 years to 65 years. The range of male participants was from 11 to 105, while the range of female participants was from 4 to 160. The gender ratio varied among the studies, with the proportion of male participants generally exceeding that of female participants in the studies that reported gender distribution.

**Fig. 2:**
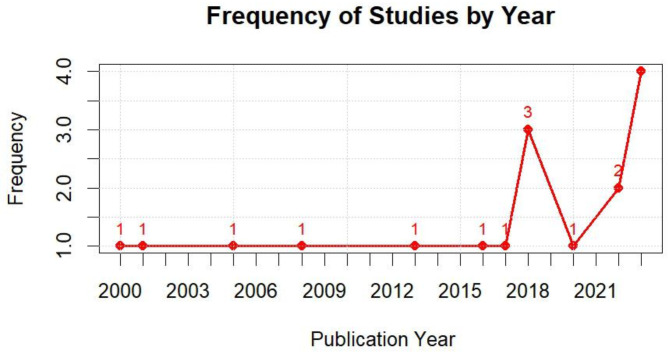
Number of published studies by year

The studies focused on various types of oral cancer, including nine studies on HNSCC, one on metastatic head and neck HNSCC, one on head and neck cancer, and six on oral squamous cell carcinoma (OSCC). The reported outcomes highlighted the prognostic and diagnostic role of the ctDNA and cfDNA in oral cancer or head and neck cancer patients. Most of the studies conducted were after 2018, as shown in the graph in Fig. 2.

Based on the main findings from the studies, there is evidence suggesting the utility of the circulating tumor DNA (ctDNA), circulating free DNA (cfDNA) as biomarkers assessing head and neck cancers, particularly for oral squamous cell carcinoma (OSCC) and head and neck squamous cell carcinoma (HNSCC). Elevated cfDNA levels have been associated with tumor size, poor prognosis, lymph node metastasis, and advanced disease stages. Additionally, ctDNA and cfDNA have potential as predictive tools for monitoring cancer progression, treatment response, recurrence risk, metastasis, and overall survival. ctDNA detection has been correlated with regional metastasis, poor progression-free survival, and certain aggressive tumor patterns, such as high-risk worst patterns of invasion and deep invasion. This demonstrates the potential of ctDNA as a powerful tool in understanding tumor behavior and guiding treatment strategies. While some studies found a positive association between higher ctDNA levels and high metabolic tumor burdens, others did not find clear links between ctDNA and overall survival. Out of the 12 studies evaluated using Newcastle-Ottawa Scale (NOS), 8 studies were determined to be of good quality, while 4 studies were classified as fair quality. (Table 2)

**Table 2:** NOS for the risk of bias and quality assessment of cohort studies (*n* = 12)

Author	Selection				Comparability	Outcome			Total score	quality
	Representativeness of the exposed cohort	Selection of the non exposed cohort	Ascertainment of exposure	outcome of interest was not present at start of study	Comparability of cohorts on the basis of the design or analysis	Assessment of outcome	follow-up long enough for outcomes to occurs	Adequacy of follow up of cohorts		
Lin et al. 2018	*	*	*	*	*	**	*	**	10	Good
Hamana et al. 2005		*	*	*	*	*	*	*	7	Good
Kakimoto et al. 2008		*	*	*	*	*	*	*	7	Good
Husain et al. 2020			*	*	*	*	*	*	6	Fair
Coulet et al. 2000			*	*	*	*	*	*	6	Fair
Taylor et al. 2023			*	*	*	*	*	*	6	Fair
Chikuie et al. 2022			*	*	*	*	*	*	6	Fair
Honoré et al. 2023			*	*	*	*	*	*	6	Fair
Kampel et al. 2023	*	*	*	*	*	**	*	*	9	Good
Egyud et al. 2018	*	*	*	*	*	**	*	**	10	Good
Mazurek et al. 2016	*	*	*	*	*	**	*	**	10	Good
Silvoniemi et al. 2023	*	*	*	*	*	*	*	*	8	Good

## Discussion

This systematic review comprehensively assessed role of the circulating tumor DNA (ctDNA) and circulating free DNA (cfDNA) as biomarkers in oral and head and neck cancers. Through a meticulous screening process, we identified 17 studies that met our inclusion criteria, providing valuable insights into the prognostic and diagnostic potential of ctDNA and cfDNA in these malignancies.

The studies included in review exhibited significant heterogeneity in terms of design, sample size, methodology, and measured outcomes. Most studies were observational, with the majority being retrospective cohort studies.

The geographic distribution of the studies was diverse, with contributions from various countries, including Taiwan, Japan, India, France, the UK, Belgium, Israel, USA, Poland, Brazil, and Finland. This multinational representation enhances the generalizability of our findings and underscores the global relevance of the topic [31]. 

Findings from the included studies highlight the potential utility of ctDNA and cfDNA as biomarkers for assessing head and neck cancers, particularly OSCC [2, 6, 14, 15, 17–19, 26] and HNSCC [25, 27–30]. Elevated cfDNA levels have been consistently associated with poor prognosis, larger tumor size, lymph node metastasis, and advanced disease stages. Furthermore, ctDNA and cfDNA have demonstrated promise as predictive tools for monitoring cancer progression, treatment response, recurrence risk, metastasis, and overall survival.

Several key observations emerged from the reviewed studies. First, ctDNA detection was correlated with certain clinicopathologic features, such as age ≤ 40, female gender, short disease duration, infiltrative tumors, and lower tumor volume, which were reported in some studies as potential indicators of tumor biology and prognosis. This suggests that ctDNA levels may serve as indicators of tumor aggressiveness and complement traditional biopsy/histopathological assessments.

Second, while some studies found a positive association between higher ctDNA levels and high metabolic tumor burden, others did not establish a clear link between ctDNA and overall survival. This discrepancy underscores the complexity of ctDNA dynamics and highlights the need for further research to clarify its prognostic significance.

Third, microsatellite analysis of serum DNA emerged as a promising approach for predicting recurrence, metastasis, and death in OSCC patients. Similarly, next-generation sequencing (NGS)-based assays for detecting TP53 mutations showed potential for predicting progression-free survival and regional metastases in HNSCC.

Out of the 18 included studies, 10 studies assessed cfDNA (cell-free DNA) as a biomarker, while 8 studies focused on ctDNA (circulating tumor DNA). Among the studies assessing cfDNA, 6 studies reported positive outcomes indicating a substantial association between cfDNA levels and various clinicopathological parameters of oral and head and neck cancers. These positive outcomes included correlations between cfDNA levels and tumor size, lymph node metastasis, late-stage disease, nodal metastasis, and clinical stages of OSCC. Additionally, cfDNA concentrations were found to significantly increase with higher cancer stages in OSCC patients. However, 1 study found no significant differences in cfDNA levels between different groups of oral malignancies, and disease progression did not correlate with changes in cfDNA levels in blood or plasma.

In contrast, among the studies focusing on ctDNA, 7 studies reported positive outcomes indicating the potential utility of the ctDNA as prognostic biomarker for oral and head and neck cancers. These positive outcomes included associations between elevated ctDNA levels and aggressive tumor characteristics, poor prognosis, recurrence, regional metastases, and disease progression. Notably, ctDNA was found to effectively predict disease progression and overall survival without the need for invasive tumor sequencing procedures in some studies. However, 1 study did not find an association between ctDNA mutations and overall survival in HNSCC patients.

Overall, the findings from our systematic review provide compelling evidence supporting the clinical utility of ctDNA and cfDNA as non-invasive biomarkers for the management of oral and head and neck cancers. These biomarkers hold promise for guiding personalized treatment strategies, monitoring disease progression, and improving patient outcomes. However, further large-scale prospective studies are necessary to validate these findings and facilitate the integration of ctDNA and cfDNA assays into routine clinical practice.

We included a total of 12 studies. In this cohort study assessment, out of the 12 studies evaluated using the Newcastle-Ottawa Scale (NOS), 8 studies were determined to be of good quality, while 4 studies were classified as fair quality. The NOS assesses the quality of the non-randomized studies based on three broad perspectives: The selection of study groups, ascertainment of either the exposure or outcome of interest and comparability of the groups [32]. A common limitation identified was within the Selection domain, specifically concerning the representativeness of the exposed cohort. This criterion assesses whether the study cohort accurately reflects the average exposed population, thereby influencing the generalizability of the findings [33]. In our analysis, eight out of twelve studies did not meet this criterion, leading to a reduction in their overall quality rating from good to fair. The lack of representativeness implies that the study populations may not accurately mirror the broader patient demographic, thereby limiting the applicability of the results to the general population. This issue is critical, as studies with non-representative cohorts may yield findings that do not generalize well to real-world settings, potentially affecting the reliability of biomarker outcomes.​ The importance of cohort representativeness in ensuring external validity has been reported in previous methodological evaluations [34]. The Newcastle-Ottawa Scale coding manual emphasizes that cohorts should be truly representative of the average in the community to ensure the applicability of study findings. Failure to achieve this representativeness can lead to biased outcomes and limit the utility of the research in wide clinical contexts.

Future studies should include diverse and representative cohorts with varying tumor stages and risk factors to improve generalizability. Using multicenter designs, consecutive sampling, and standardized biomarker detection methods can minimize selection bias and enhance the reliability of ctDNA and cell-free DNA biomarkers in OSSC.

Incorporating circulating tumor DNA (ctDNA) and cell-free DNA (cfDNA) into clinical practice holds promise for personalized medicine, especially in treatment monitoring. These biomarkers enable non-invasive, real-time assessment of tumor dynamics, aiding early detection of resistance and disease progression [35]. However, technical challenges like varying sensitivity of detection methods like digital polymerase chain reaction (dPCR) and next-generation sequencing (NGS) and lack of standardized protocols hinder their clinical utility. Standardizing pre-analytical and analytical procedures will improve their reliability, paving the way for better personalized treatment strategies [36]. 

Other biomarkers and diagnostic techniques could complement ctDNA and cfDNA in oral cancer diagnosis. Conventional markers like p16 help assess HPV-related tumors, while imaging methods such as PET-CT and MRI offer detailed structural and functional information. Combining these approaches with ctDNA and cfDNA could provide a more comprehensive evaluation of tumor behavior, improving diagnostic accuracy, treatment monitoring, and prognosis [37]. 

Strengths of this study include adherence to stringent guidelines outlined by the Cochrane handbook and reporting in accordance with the PRISMA checklist. A comprehensive search was conducted across major databases, and the study addressed a focused research question.

However, this study is not without limitations. The included studies encompass a diverse population, with some focusing on oral squamous cell carcinoma (OSCC) and others on head and neck squamous cell carcinoma (HNSCC). Due to high clinical heterogeneity and varying effect sizes, pooling the results was not feasible. Additionally, the studies exhibit variability in sample sizes and methodologies.

## Conclusion

Within the limitations of this study, it can be concluded that ctDNA and cfDNA exhibit promising prognostic and diagnostic potential in both oral squamous cell carcinoma (OSCC) and head and neck squamous cell carcinoma (HNSCC). Elevated cfDNA levels are associated with poor prognosis, lymph node metastasis, larger tumor size, and advanced disease stages across multiple studies. Similarly, ctDNA functions as a predictive tool for monitoring cancer progression, treatment response, recurrence risk, metastasis, and overall survival.

## Data Availability

The datasets used and/or analysed during the current study are available from the corresponding author on reasonable request.
